# Association between soft drink consumption types and risk of lung cancer and all-cancer: A prospective study of PLCO data

**DOI:** 10.7555/JBR.36.20220135

**Published:** 2022-11-20

**Authors:** Dongfang You, Hongyang Xu, Xin Chen, Jiawei Zhou, Yaqian Wu, Yingdan Tang, Zhongtian Wang, Yang Zhao, Fang Shao

**Affiliations:** 1 Department of Biostatistics, School of Public Health, Nanjing Medical University, Nanjing, Jiangsu 211166, China; 2 Department of Critical Care Medicine, Wuxi People's Hospital Affiliated to Nanjing Medical University, Wuxi, Jiangsu 214023, China; 3 China International Cooperation Center for Environment and Human Health, Center for Global Health, Nanjing Medical University, Nanjing, Jiangsu 211166, China; 4 The Center of Biomedical Big Data and the Laboratory of Biomedical Big Data, Nanjing Medical University, Nanjing, Jiangsu 211166, China; 5 Jiangsu Key Lab of Cancer Biomarkers, Prevention and Treatment, Collaborative Innovation Center for Cancer Personalized Medicine, Nanjing Medical University, Nanjing, Jiangsu 211166, China

**Keywords:** soft drink, diet/sugar-free drink, lung cancer, all-cancer, PLCO trial

## Abstract

Diet/sugar-free soft drinks are considered to be healthier than regular soft drinks. However, few studies have examined the relationship between the types of soft drinks (regular and diet/sugar-free) and lung cancer (LC)/all-cancer (AC) risk. In this study, we comprehensively assessed the influence of the type of soft drink consumption on LC/AC risk based on the Prostate, Lung, Colorectal, and Ovarian (PLCO) Cancer Screening Trial. Multivariable Cox proportional hazards and competing risks Fine-Gray regression models adjusted for relevant confounders were used to estimate hazard ratios (HRs) and subdistribution HRs for different types of soft drink consumption. In the PLCO population, female subgroup, and the ever/current smoker subgroup, consumption of both regular and diet soft drinks was associated with a significantly reduced risk of LC compared with no soft drinks at all. For the non-lung cancer (NLC) risk, consumption of only diet soft drinks had a significant positive association for the total population and female subgroup. Based on our findings, it was suggested that partial replacement of regular soft drinks with diet soft drinks might be beneficial to LC prevention, especially for females and ever/current smokers. Additionally, completely replacing regular soft drinks with diet soft drinks might be detrimental to NLC prevention, especially for females.

## Introduction

Cancer is the leading cause of death, and lung cancer (LC) is the leading cause of cancer deaths globally ^[ 1] ^. It was estimated that LC has caused 131 880 deaths in 2021 in the United States ^[ 2]^ and cancer became the world's second-leading cause of diet-related death in 2017 ^[ 3] ^. Risk factors of LC are mainly smoking, genetic, and other environmental factors ^[ 4] ^. Some studies have also reported an association between dietary factors and cancer risk ^[ 5] ^. Plant-based foods, including fruits and vegetables, are categorized as protective factors for cancer, while red meat, processed meat, and alcoholic drinks are categorized as risk factors ^[ 6] ^. Coffee, tea, and milk consumption are also associated with LC ^[ 7– 8] ^. However, few studies have examined the relationship between the types of soft drinks (regular and diet/sugar-free) and LC/all-cancer (AC) risk.

Soft drinks mainly include soda, pop, syrup, or other sugary carbonated or non-carbonated non-alcoholic beverages ^[ 9] ^. Previously published systematic reviews, meta-analyses, and cohort studies showed that the consumption of soft drinks (regular and diet) affected the morbidity and mortality of several diseases ^[ 9– 17] ^, including type 2 diabetes, metabolic syndrome, and coronary heart disease. Although less tasty than regular soft drinks, diet soft drinks are preferred by consumers because they contain less sugar and are considered healthier than regular soft drinks. However, some studies have suggested that consumption of diet/sugar-free soft drinks or artificially sweetened beverage is a risk factor for all-cause and cardiovascular disease (CVD) death ^[ 10, 16– 18] ^. It would therefore be interesting to study the relationship between the types of soft drink consumption and the incidence of LC and AC.

In the present study, we performed a comprehensive analysis of prospectively collected data from the Prostate, Lung, Colorectal, and Ovarian (PLCO) Cancer Screening Trial to investigate the role of soft drink consumption type in LC/AC risk. In the PLCO population, consumption of both regular and diet soft drinks was associated with a significantly reduced risk of LC compared with no soft drinks at all. For non-lung cancer (NLC) risk, consumption of only diet soft drinks had a significant positive association. According to our findings, it was suggested that partial replacement of regular soft drinks with diet soft drinks might be beneficial to LC prevention, and completely replacing regular soft drinks with diet soft drinks might be detrimental to NLC prevention.

## Materials and methods

### Study design and participants

The PLCO trial is a randomized, controlled, two-armed trial conducted by the National Cancer Institute ^[ 19– 20] ^. PLCO trial was designed to evaluate the efficacy of selected cancer screening methods in reducing mortality from PLCO cancers. From 1993 to 2001, the PLCO trial enrolled 154 897 eligible participants aged 55 to 74 years from 10 study centers in the United States. The PLCO trial had two randomized arms, including an intervention arm and a control arm. Participants in the control arm received usual care, while those in the intervention arm were screened for PLCO cancers during the first 3–4 years. All participants were followed up until they died or until December 31, 2009, whichever came first. Both arms received more than 10 years of follow-up after enrollment (median follow-up time 11.3 years). The primary recruitment strategy was mass mailing to the general population living near the screening center. Patients who had a history of some type of cancer within the PLCO and were currently undergoing cancer treatment or post-pneumonectomy were excluded, and all other responders were included in the study.

**Figure 1:**
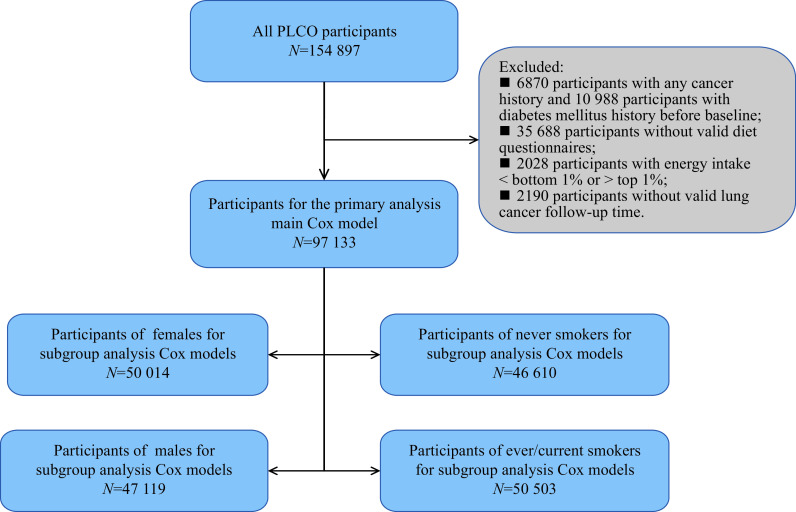
Flowchart of study participants for LC outcomes.

The self-administered baseline questionnaire (BQ), dietary history questionnaire (DHQ), and supplementary questionnaire (SQX) completed by participants provided information on related factors for this study ^[ 19– 20] ^. Data from the above questionnaires were merged based on the PLCO ID numbers for subsequent analyses. Totally, there were 154 897 observations in the PLCO dataset. During quality control, observations of any pre-baseline history of cancer and diabetes mellitus, invalid data of DHQ, absence from follow-up, and extreme energy consumption (top 1% or bottom 1%) were excluded. A total of 2190 observations without valid follow-up time for LC were also excluded. The final remaining data set for subsequent analyses with LC outcomes consisted of 97 133 observations. The same quality control process was also conducted for AC outcomes, and the final data set consisted of 92 997 observations. The flow charts of this study are shown in ***Fig. 1*** and *Supplementary Fig. 1* (available online) for LC and AC outcomes, respectively. The median follow-up time was 8.9 years for both LC and AC outcomes.

### Data collection

Upon entering the PLCO trial, participants completed the BQ about demographics ( *e.g.*, age, sex, education, ethnicity), lifestyle ( *e.g.*, smoking and alcohol consumption behavior), family history of cancers, personal health history, and past screenings. All participants received an annual study update questionnaire asking if any cancers had been diagnosed in the past year ^[ 19– 23] ^. DHQ was a food frequency questionnaire with 156 questions, providing a variety of dietary nutrition data on consumption of various foods, drinks, and supplements. SQX provided additional background information, including factors related to physical activity.

DHQ provided the daily gram of consumption of regular and diet/sugar-free soft drinks which was calculated using the detailed analysis file output by DietCalc based on the coded responses to soft drink consumption frequency and serving size questions ^[ 19– 20] ^. Soft drinks defined in DHQ include soft drinks, soda, and pop. The consumption of soft drink was divided into the following four types: 1, "None": No regular drinks or diet/sugar-free soft drinks; 2, "Regular only": Only regular soft drinks; 3, "Diet only": only diet/sugar-free soft drinks; 4, "Both": Both regular and diet/sugar-free soft drinks.

### Confirmation of lung cancer and all-cancer

As previously mentioned, the incidence of LC was primarily determined by chest radiography and annual study update questionnaire. The annual study update questionnaires were mailed to participants about the cancer diagnosis in the previous year. Participants who did not respond to the questionnaires would be contacted by repeat mailings or phone calls ^[ 22– 24] ^. Follow-up was confirmed by regular contact with state cancer registries and death registries supplemented by physician reports.

All LC cases were confirmed by pathology. The follow-up began one year after completion of DHQ until the participant was diagnosed with cancer, withdrew from the trial, died from any cause, or completed a 10-year follow-up, whichever came first ^[ 19– 23] ^. Similarly, AC cases were confirmed by medical record abstraction, including PLCO diagnosis, other cancer forms, and annual study update forms ^[ 19– 20] ^.

### Statistical analysis

The primary analysis was the relationship between soft drink consumption types and LC. The secondary analysis was the relationship between soft drink consumption types and AC. Analyses of the whole population and subgroups including males, females, never smokers, and ever/current smokers were considered.

As mentioned before, the consumption of soft drink was divided into four types: "None", "Regular only", "Diet only" and "Both". Continuous variables such as age and total energy intake were expressed as the means and standard deviations (SDs), and the categorical data such as sex, smoking status, and family history of LC/AC were expressed as numbers and percentages. Kruskal Wallis test was used for continuous variables and Chi-square test was used for categorical variables to compare the demographic characteristics of the four soft drink consumption types and LC/AC strata.

Multivariable Cox regression models were used to estimate hazard ratios (HRs) and 95% confidence intervals (CIs) for LC or AC. Potential confounders were adjusted in the models, including age, sex (not for male and female subgroups), race, arm, center, family history of LC/AC, the education level (less than 12 years old, post-high school training other than college, and college and above), body mass index (BMI) types at baseline, smoking status (not for never and ever/current smokers subgroups), physical activity status, daily energy consumption, red meat, fruits, vegetables and coffee, alcohol consumption, and estrogen use (the female subgroup only) ^[ 5, 8, 10, 22] ^.

The AC outcomes were further subdivided into LC and NLC outcomes. Cause-specific Cox and competing risks Fine-Gray regression models were performed accordingly. Potential confounders shown above were adjusted in the models.

Cumulative incidences of LC/AC outcomes stratified by soft drink consumption types were estimated and log-rank tests were performed. Cause-specific and competing risks cumulative incidences of LC/NLC outcomes by soft drink consumption types were also estimated. The log-rank test and Gray's test were performed for LC and NLC, respectively.

All the statistical analyses were performed using R software ^[ 25] ^, version 4.2.0 with R packages "cmprsk" ^[ 26] ^, "survival" ^[ 27– 28] ^, and "tableone" ^[ 29] ^. *P*<0.05 was considered statistically significant.

## Results

### Demographic characteristics across soft drink consumption types

Simplified descriptive characteristics of eligible PLCO participants with LC outcomes according to the types of soft drink consumption are presented in ***Table 1***, and complete descriptive characteristics of the participants with LC and AC outcomes are presented in ***Supplementary Table 1– 4*** (available online). All the demographic characteristics showed significant association with soft drink consumption types ( *P*<0.001). All the demographic characteristics, including soft drink consumption types, showed significant association with LC/AC outcomes ( *P*<0.001) except for the "Arm" ( *P*=0.868) and "Alcohol consumption" ( *P*=0.569) with LC outcome.

**Table 1:** Characteristics at baseline with LC outcomes across soft drink consumption types

Variables	Soft drink consumption types			
	None ( *N*=8643)	Regular only ( *N*=37019)	Diet only ( *N*=35557)	Both ( *N*=15914)
LC cases ( *n* [%])				
No	8470 (98.0)	36362 (98.2)	35070 (98.6)	15714 (98.7)
Yes	173 (2.0)	657 (1.8)	487 (1.4)	200 (1.3)
LC type ( *n* [%])				
Non-small cell	147 (1.7)	566 (1.5)	415 (1.2)	175 (1.1)
Small cell	26 (0.3)	91 (0.2)	72 (0.2)	25 (0.2)
Missing	8470 (98.0)	36362 (98.2)	35070 (98.6)	15714 (98.7)
Age (years, mean [SD])	64.01 (5.41)	62.47 (5.33)	61.82 (5.10)	62.69 (5.28)
Sex ( *n* [%])				
Male	5382 (62.3)	15916 (43.0)	21623 (60.8)	7093 (44.6)
Female	3261 (37.7)	21103 (57.0)	13934 (39.2)	8821 (55.4)
Race ( *n* [%])				
White	7841 (90.7)	33097 (89.4)	33755 (94.9)	14383 (90.4)
Black	175 (2.0)	1651 (4.5)	522 (1.5)	472 (3.0)
Hispanic	137 (1.6)	584 (1.6)	372 (1.0)	212 (1.3)
Asian	424 (4.9)	1445 (3.9)	740 (2.1)	719 (4.5)
Others	66 (0.8)	242 (0.7)	168 (0.5)	128 (0.8)
BMI (kg/m ^2^, *n* [%])				
Underweight (0–18.5)	152 (1.8)	275 (0.7)	150 (0.4)	63 (0.4)
Normal (18.5–25)	4027 (46.6)	13783 (37.2)	10503 (29.5)	4472 (28.1)
Overweight (25–30)	3076 (35.6)	15892 (42.9)	15082 (42.4)	7326 (46.0)
Obesity (≥30)	1258 (14.6)	6586 (17.8)	9387 (26.4)	3839 (24.1)
Missing	130 (1.5)	483 (1.3)	435 (1.2)	214 (1.3)
Education ( *n* [%])				
Less than 12 years	2265 (26.2)	11439 (30.9)	9660 (27.2)	4479 (28.1)
Post high school training other than college	1069 (12.4)	4936 (13.3)	4381 (12.3)	2074 (13.0)
College and above	5297 (61.3)	20568 (55.6)	21445 (60.3)	9327 (58.6)
Missing	12 (0.1)	76 (0.2)	71 (0.2)	34 (0.2)
Smoking status ( *n* [%])				
Never	4180 (48.4)	18277 (49.4)	16135 (45.4)	8018 (50.4)
Ever	814 (9.4)	4599 (12.4)	2473 (7.0)	1102 (6.9)
Current	3646 (42.2)	14135 (38.2)	16943 (47.7)	6791 (42.7)
Missing	3 (0.0)	8 (0.0)	6 (0.0)	3 (0.0)
Family history of LC ( *n* [%])				
No	7521 (87.0)	31978 (86.4)	30697 (86.3)	13836 (86.9)
Yes	871 (10.1)	3761 (10.2)	3855 (10.8)	1563 (9.8)
Possibly	199 (2.3)	1009 (2.7)	712 (2.0)	382 (2.4)
Missing	52 (0.6)	271 (0.7)	293 (0.8)	133 (0.8)
LC: lung cancer; BMI: body mass index; SD: standard deviation.				

### Association between soft drink consumption types and lung cancer risk

**Figure 2:**
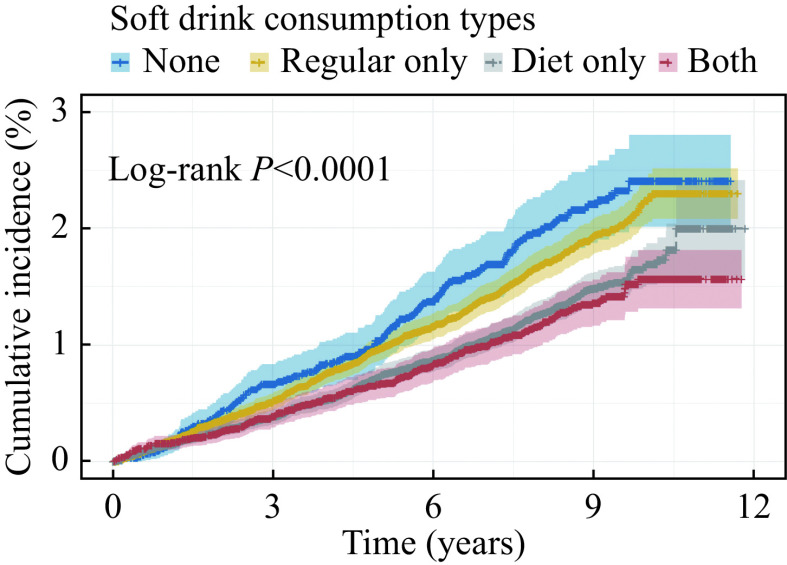
Cumulative incidence of LC stratified by soft drink consumption types.

The cumulative incidence of LC stratified by soft drink consumption types is shown in ***Fig. 2***. Significant differences in the estimated cumulative incidence were demonstrated since the *P-*value of the log-rank test is less than 0.001 for LC risk.

**Figure 3:**
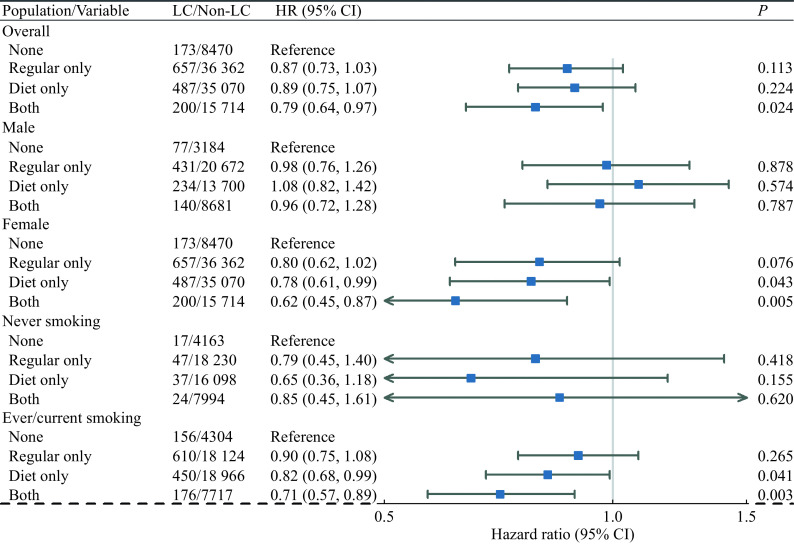
Forest plot of estimated adjusted HRs for soft drink consumption types with LC outcomes for different populations.

Results of adjusted HRs and corresponding CIs for soft drink consumption types estimated by main Multivariable Cox regression models for LC outcomes are shown in ***Fig. 3*** and *Supplementary Table 5* (available online). For the total population, soft drink consumption had a protective effect with statistically significant adjusted HRs for the "Both" type with the "None" type as reference (hazard ratio [HR]=0.79, 95% confidence interval [CI]: 0.64–0.97, *P*=0.024). Both regular and diet/sugar-free soft drink consumption were associated with a significant reduction in LC risk. A decreasing trend of HRs for different soft drink consumption types was identified with the HRs for "Diet only", "Regular only" and "Both" types sequentially.

Similar results were shown in the female and ever/current smoking subgroups. Soft drink consumption had protective effects. A decreasing trend of HRs for different soft drink consumption types was identified with the HRs for "Regular only", "Diet only", and "Both" types sequentially. For the female subgroup, "Diet only" and "Both" types had statistically significant adjusted HRs with the "None" type as reference ("Diet only", HR=0.78, 95% CI: 0.61–0.99, *P*=0.043; "Both", HR=0.62, 95% CI: 0.45–0.87, *P*=0.005). For the ever/current smoking subgroup, "Diet only" and "Both" types had statistically significant adjusted HRs ("Diet only", HR=0.82, 95% CI: 0.68–0.99, *P*=0.041; "Both", HR=0.71, 95% CI: 0.57–0.89, *P*=0.003). Diet/sugar-free soft drink consumption was associated with a significant reduction in LC risk and drinking both regular and diet soft drinks had the lowest risk. For male and never-smoking status subgroups, no statistically significant results were found for soft drink consumption.

### Association between soft drink consumption types and all-cancer risk

The cumulative incidence of AC stratified by soft drink consumption types is shown in ***Supplementary Fig. 2*** (available online). Similar to the LC outcomes, there were significant differences in the estimated cumulative incidence since the *P-*value of the log-rank test was less than 0.001 for AC.

Multivariable Cox regression models for the AC outcomes were also conducted to discover the association between soft drink consumption and the AC outcomes. The results of estimated HRs for soft drink consumption types are demonstrated in ***Supplementary Fig. 3*** (available online) and ***Supplementary Table 6*** (available online). Although there were as similar declining trends of HRs for different soft drink consumption types as the previous primary analysis results of LC outcomes, no statistically significant results were found in the total population and subgroups of males, females, never, and ever/current smokers.

### Association between soft drink consumption types and competing risks of lung cancer/non-lung cancer

**Figure 4:**
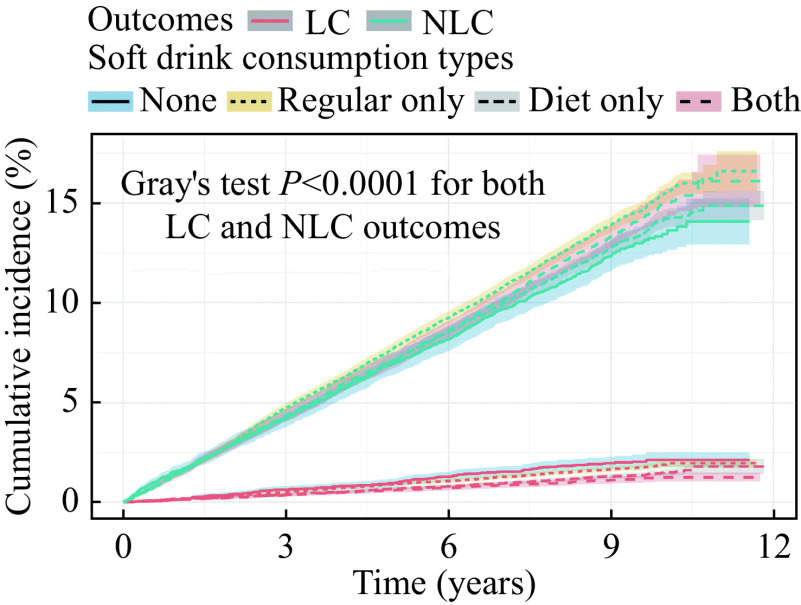
Competing risks cumulative incidences of LC and NLC outcomes stratified by soft drink consumption types.

The AC outcomes were subdivided into LC and NLC outcomes. The cause-specific and competing risks cumulative incidences of LC and NLC stratified by soft drink consumption types are shown in ***Fig. 4*** and ***Supplementary Fig. 4*** and ***5*** (available online). The cause-specific and competing risks cumulative incidences were similar. Similar to the previous results of LC and AC outcomes, there were significant differences in the estimated cause-specific and competing risks cumulative incidence since the *P-*values of the log-rank and Gray's tests were less than 0.001 for both LC and NLC.

**Figure 5:**
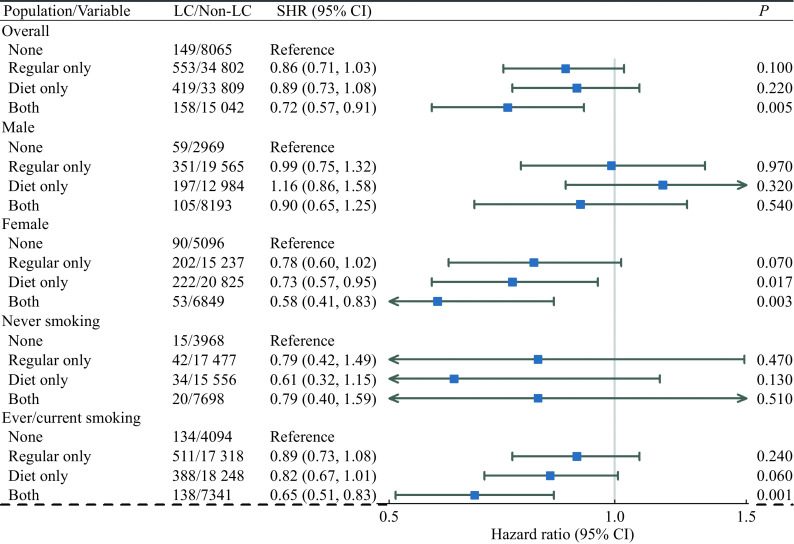
Forest plot of estimated adjusted subdistribution HRs for soft drink consumption types with LC outcomes for different populations.

**Figure 6:**
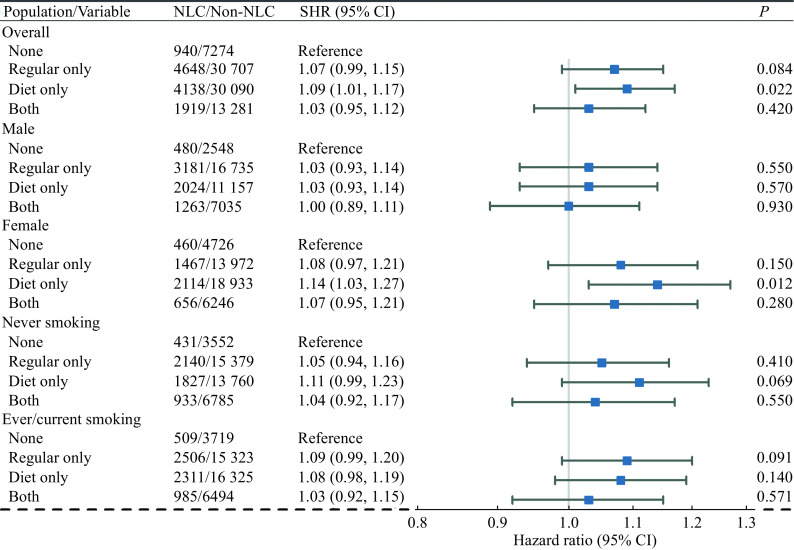
Forest plot of estimated adjusted subdistribution HRs for soft drink consumption types with NLC outcomes for different populations.

Cause-specific multivariable Cox regression models and competing risks Fine-Gray models were conducted to discover the association between soft drink consumption and the LC and NLC outcomes. The results of estimated cause-specific hazard ratios (CHRs) and competing risks subdistribution hazard ratios (SHRs) for soft drink consumption types are demonstrated in ***Fig. 5*** and ***6***, ***Supplementary Fig. 6*** and ***7*** (available online) and ***Supplementary Table 7*** and ***8*** (available online). Results of corresponding CHRs and SHRs were similar. CHRs and SHRs for LC were consistent with the results of HR for LC outcomes in the primary analysis shown before.

For the total population, soft drink consumption had a protective effect with statistically significant adjusted CHR and SHR for the "Both" type with the "None" type as reference for LC (CHR=0.71, 95% CI: 0.57–0.90, *P*=0.004; SHR=0.72, 95% CI: 0.57–0.91, *P=*0.005). Both regular and diet/sugar–free soft drink consumptions were associated with a significant reduction in LC risk. For NLC, soft drink consumption had a harmful effect with statistically significant adjusted CHR and SHR for the "Diet only" type with the "None" type as reference (CHR=1.09, 95% CI: 1.01–1.07, *P=*0.025; SHR=1.09, 95% CI: 1.01–1.07, *P=*0.022). Decreasing trends of CHRs and SHRs for different soft drink consumption types for both LC and NLC outcomes were identified for "Diet only", "Regular only" and "Both" types sequentially. These trends were consistent with the results of the previous primary analysis of LC outcomes and the secondary analysis of AC outcomes.

Similar results were shown in the female and ever/current smoking subgroups for LC. Soft drink consumption had protective effects. A decreasing trend of HRs for different soft drink consumption types was identified with the HRs for "Regular only", "Diet only", and "Both" types sequentially. For the female subgroup, "Diet only" and "Both" types had statistically significant adjusted HRs with the "None" type as reference ("Diet only", CHR=0.74, 95% CI: 0.57–0.95, *P=*0.018, SHR=0.73, 95% CI: 0.57–0.95, *P=*0.017; "Both", CHR=0.59, 95% CI: 0.42–0.83, *P=*0.003, SHR=0.58, 95% CI: 0.41–0.83, *P=*0.003). For the ever/current smoking subgroup, "Diet only" type had statistically significant adjusted CHR, and "Both" type had significant adjusted CHR and SHR ("Diet only", CHR=0.82, 95% CI: 0.67–1.00, *P=*0.048; "Both", CHR=0.65, 95% CI: 0.51–0.83, *P=*0.001, SHR=0.65, 95% CI: 0.51–0.83, *P=*0.001). Diet/sugar–free soft drink consumption was associated with a significant reduction in LC risk and drinking both regular and diet soft drinks had the lowest risk. For male and never–smoking status subgroups, no statistically significant results were found for soft drink consumption.

For NLC, similar results were shown in the female subgroup. Soft drink consumption had a harmful effect with statistically significant adjusted CHR and SHR for the "Diet only" type with the "None" type as reference (CHR=1.14, 95% CI: 1.03–1.27, *P=*0.014; SHR=1.14, 95% CI: 1.03–1.27, *P=*0.012). For male, ever/current smoking, and never smoking subgroups, no statistically significant results were found for soft drink consumption.

### Sensitivity analysis

Sensitivity analyses of small/non-small cell LC (SCLC/NSCLC) types were performed by using the main multivariable Cox models with adjusted variables. Results are shown in *Supplementary Fig. 8* and ***9*** (available online), which were similar to the results obtained from the overall LC outcomes. A decreasing trend of HRs for different soft drink consumption types was shown. Soft drink consumption had a protective effect. For NSCLC outcomes, the "Both" types had statistically significant adjusted HRs with "None" type as a reference in the female subgroup (HR=0.66, 95% CI: 0.46–0.93, *P=*0.019) and ever/current smoking subgroup (HR=0.71, 95% CI: 0.56–0.91, *P*=0.006). However, for SCLC outcomes, no statistically significant results of soft drink consumption were investigated due to the limited cases.

The protective effects of soft drink consumption types were demonstrated. Compared with no soft drink consumption, people having both regular and diet soft drinks were inversely associated with LC risk for females and ever/current smoking subgroups. These findings were not only consistent with the primary analysis of the LC outcomes, but also suggested that soft drink consumption types might have different protective effects on LC types.

## Discussion

In this large prospective study of the association between types of soft drink consumption and LC/AC risks, diet/sugar-free soft drink consumption was found to be negatively associated with LC risk, particularly in females and ever/current smokers. People who consumed both regular and diet soft drinks had the lowest risk of LC compared with those not drinking any soft drinks, especially females and ever/current smokers. These findings suggested that taking diet soft drinks as a partial alternative to regular soft drinks might be helpful in preventing LC. Although the overall trend was similar, there were no similarly significant results for AC risk. The consumption of only diet soft drinks was positively associated with the NLC risk, particularly in females. In sensitivity analyses, the overall trend was similar across different LC types although there was a lack of statistical significance in some subgroups.

Previous studies have discussed the relationship between the cancer mortality and soft drink consumption ^[ 16– 17] ^. There were no significant associations for either regular or diet soft drinks with the cancer mortality ^[ 16– 17] ^. In our secondary analysis of AC outcomes, there was no significant association between the incidence of AC and the type of soft drink consumption. Our result was consistent with previous studies. However, in our analysis of subdivided LC and NLC outcomes, significant associations were found. This suggested that the type of soft drink consumption might have different associations with different cancer incidences. However, this was not sufficient for causal inference, and the association needed to be further validated. Therefore, our findings should be interpreted with caution. If these associations were confirmed, it would be meaningful to investigate the mechanisms behind them.

Refined carbohydrates from sugar-sweetened beverages are associated with an increased risk of LC ^[ 22] ^. Therefore, it is necessary to continue advocating strict restrictions on the consumption of sugar-sweetened beverages. A partial replacement of regular soft drinks with diet soft drinks might be beneficial to overall health and have the potential to be useful in LC prevention. The preference for soft drinks indicates that soft drinks might have some positive effects on people, potentially emotionally or mentally ^[ 11, 15, 30] ^. However, regular soft drinks are sugar-sweetened beverages, which are not good for one's health ^[ 9] ^. Switching to diet soft drinks might retain the positive effects of regular soft drinks on people to some extent, while reducing the negative effects of sugar intake on health. Since there was a difference in taste between diet and regular soft drinks, completely replacing regular soft drinks with diet soft drinks might not retain the positive effects of regular soft drinks and might be detrimental to NLC prevention. Different results of the subgroup analyses suggested different effects on the types of soft drink consumption in different populations. Women and ever/current smokers were more likely to prevent LC by partially replacing regular soft drinks with diet soft drinks. For NLC risk, women might have an increased risk due to completely replacing regular soft drinks with diet soft drinks. Overall, this study was only an exploratory analysis of the effect of soft drink consumption types on LC and AC risks. Thus, our findings should be interpreted with caution. Prospective cohort studies with large samples of follow-up and more comprehensive inclusion of factors related to soft drink consumption are still needed to confirm the results of this study.

The present study was an analysis of prospective cohort data from the advantageous PLCO cancer screening trial ^[ 22] ^. Therefore, the main strengths of our study included its prospective design, large-sample size, and long-term follow-up. It provided more convincing evidence for causal inference than case-control studies. The abundant data from the PLCO trial enabled us to adjust for a broad range of potential confounders (including diet-related variables) and examine the potential influence of reverse causality. Although other prospective cohort studies, such as the UK Biobank ^[ 10, 31] ^, had larger total sample sizes, the proportions of lung cancer cases were much smaller, so PLCO had significant strength. Moreover, this study was the first to comprehensively evaluate the association between soft drink consumption types ("None", "Regular only", "Diet only" and "Both") and LC/AC incidence with significant HR results of Cox and competing risks Fine-Gray models with adjusted confounders. The mechanisms behind these significant associations would be meaningful to study if they were confirmed.

Nevertheless, this study had some limitations, so our findings should be interpreted with caution. Firstly, Dietary information was collected in the form of cross-sectional questionnaires in the prospective PLCO trial. Long-term dietary habits might not be correctly reflected. Secondly, self-reported information by the baseline and food frequency questionnaires were usually prone to bias ^[ 32]^ because participants might feel stressed. For example, smoking status was obtained by self-reporting, so the accuracy was uncertain, which might affect the results of Cox and Fine-Gray models with adjusted confounders ^[ 33] ^. Thirdly, the number of cancer cases might be insufficient to obtain reliable results by various subgroup analyses. Fourthly, despite adjustment for a range of potential confounders, this study could not eliminate residual confounders and thus verify the cause of the observed associations. Finally, our results were not sufficient for causal inference, and the findings needed to be further validated. In conclusion, this prospective analysis of the PLCO trial showed that after adjusting for relevant confounders, people who consumed both regular and diet soft drinks had a reduced risk of LC compared with non-drinkers, suggesting that partial replacement of regular soft drinks with diet soft drinks might be beneficial to LC prevention, especially for females and ever/current smokers. On the other hand, those who consumed only diet soft drinks had an increased risk of NLC, suggesting that completely replacing regular soft drinks with diet soft drinks might be detrimental to NLC prevention, especially for females.
